# Implementation outcomes and strategies of a peer recovery coach program: findings from a qualitative assessment in the U.S. South, 2024–2025

**DOI:** 10.1186/s13722-025-00624-4

**Published:** 2025-12-18

**Authors:** Umedjon Ibragimov, Nicholas A. Giordano, Sneha Amaresh, Tatiana Getz, Tatiana Matuszewski, Alaina R. Steck, Yan Li, Eliot H. Blum, Jessica Tuttle, Hardik Pipalia, Hannah L. F. Cooper, Joseph E. Carpenter

**Affiliations:** 1 Center for Population Sciences and Health Equity, College of Nursing, Florida State University, 2010 Levy Avenue, Innovation Park, Building B, Suite 3600, Tallahassee, FL 32310 USA; 2 Nell Hodgson Woodruff School of Nursing, Emory University, Atlanta, Georgia; 3 Department of Behavioral, Social and Health Education Sciences, Rollins School of Public Health, Emory University, Atlanta, Georgia; 4 Department of Emergency Medicine, School of Medicine, Emory University, Atlanta, Georgia; 5 Georgia Department of Public Health, Atlanta, Georgia; 6 Aniz, Inc. Holistic Harm Reduction Integrated Care Clinic, Atlanta, Georgia; 7 Georgia Poison Center, Atlanta, Georgia

## Abstract

**Introduction:**

Successful implementation of peer recovery coach (PRC) programs may help improve linkage to services and clinical outcomes for emergency department (ED) patients with substance use disorder (SUD). However, literature on implementation outcomes and strategies of PRC programs is limited. We conducted a qualitative assessment of implementation outcomes and strategies for an ED-based PRC program in Atlanta, Georgia.

**Methods:**

We conducted qualitative interviews with 27 program participants (ED patients with SUD served by PRC program) and 29 service providers and partners (peer recovery coaches, ED physicians and staff, SUD treatment and other service providers) in October 2023 – March 2025. We transcribed audio-recordings and analyzed data using rapid qualitative analysis approach mapping emerging themes to Proctor’s model of implementation outcomes and Leeman et al.’s implementation strategies framework.

**Results:**

We identified two major themes related to implementation outcomes: (1) *PRC program acceptability* (patients’ positive interactions with PRCs) and (2) *appropriateness* (sub-themes include: successful linkage to community services; PRC program as an important resource for patients; added value of PRC team; no negative impact on ED workflow). Themes related to implementation strategies include (1) *streamlined communication between PRC and ED teams* (direct communication via electronic medical records system, single contact phone number, informing ED service providers of clinical and program outcomes), (2) *addressing barriers to community-based services* (preparing patient’s medical documentation, insurance, transportation to community services); (3) *supportive supervision of PRCs* (addressing daily and long-term issues through regular meetings; limiting caseload; and providing orientation, on-job training and mental health support) and (4) *addressing telehealth implementation challenges* (ensuring access to electronic medical records system).

**Conclusion:**

This study outlines key implementation outcomes and strategies for PRC programs, offering practical guidance for successful ED-based PRC program implementation.

**Supplementary Information:**

The online version contains supplementary material available at 10.1186/s13722-025-00624-4.

## Introduction

Substance use and related challenges continue to have deadly consequences in the US, with more than 80,000 lives lost in 2024 [1, 2]. While the number of overdose deaths has dramatically declined by almost 29% since its peak in mid- 2023, it is 69% higher compared to a 12-month period ending in January 2015 [2]. Drug overdoses remain the leading cause of death among adults aged 18–44 [3]. Despite a decline in overall overdose deaths, there has been a rapid increase in stimulant misuse that is coinciding with a rise in fentanyl-related deaths involving the co-use of stimulants [4, 5]. As such, rapid, multi-pronged, and tailored efforts are needed to meet the care needs of individuals living with substance use disorders seeking emergent care.

Emergency departments (EDs) often serve as the first point of contact for individuals experiencing overdose and other acute crises related to substance use disorder (SUD). Between 2020 and 2021, according to the latest available data, the rate of ED visits with the primary diagnosis of SUD was 103.8 per 100,000 population, a 40% increase compared to 2018–2019 [6]. The traditional focus of EDs on immediate stabilization of patients rather than addressing their longer-term recovery needs has left a gap in service for individuals with SUDs.

In recent years, peer recovery coach (PRC) programs have emerged as an innovative solution to address the complex needs of individuals with SUDs within the ED setting. Peer recovery coaches - individuals with lived experience of substance use and recovery - provide emotional and practical guidance to patients with SUD and link them to hospital and community-based harm reduction, treatment and recovery services in a non-judgmental manner [7–9]. These programs have shown promise in improving access to and utilization of SUD treatment and other services and reducing repeated ED visits for overdose treatment [10–14].

ED-based PRC programs are relatively novel interventions, and knowledge about their effective implementation is still evolving. Despite the growing evidence of their success, challenges related to program integration, sustainability, and workforce development remain significant barriers to widespread implementation [15]. Most implementation science literature on ED-based PRC programs describes the process, barriers and facilitators for program implementation [see, e.g. 8]. However, there is limited knowledge on PRC program implementation outcomes and strategies to improve them. Even less is known about telehealth or hybrid PRC program implementation [16]. To address this gap, this qualitative study explores implementation outcomes and strategies of an ED-based PRC program combining in-person and telehealth components in a major hospital emergency department. The study is guided by Proctor et al.’s [17] model of implementation outcomes as well as Leeman et al.’s [18] framework grouping strategies into five categories: dissemination, implementation process, capacity building, integration and scale-up strategies.

## Methods

### Overview and design

This exploratory qualitative study was conducted as part of the implementation process assessment of Linking Individuals Needing Care for Substance Use Disorders in Urban Emergency Departments to Peer Coaches (LINCS UP) program at Grady Memorial Hospital, Atlanta, Georgia (National Clinical Trial identification number 05847621). The program started in April 2023 and is implemented by six PRCs into two teams—one team is stationed at Grady and serves patients in-person, while the other engages patients via telehealth. Both in-person and telehealth teams engage patients with SUD at Grady’s ED and link them to SUD treatment and recovery support services provided outside of the hospital, following up with discharged patients over the phone. In-person team members have physical access to the hospital, meet care team members face-to-face, and have access to the hospital’s electronic medical record system (EMR). Conversely, telehealth team members are stationed outside of the hospital and hosted by LINCS UP program partner – Georgia Council for Recovery, a local recovery community organization. Telehealth arm patients connect with PRCs via teleconferencing platform using a handheld device (iPad) brought to the patients by LINCS UP staff and do not have access to the EMR due to hospital policies surrounding EMR access for outside employees.

From October 2023 to March 2025, we interviewed the LINCS UP program participants (patients) as well as key contributors to program implementation: LINCS UP PRC team, Grady ED physicians and staff, as well as representatives of SUD service providers partnering with LINCS UP in Metro Atlanta, Georgia.

### Research team characteristics and reflexivity

This study team comprises public health researchers, healthcare professionals, and public health students. The senior author (JC), an emergency department physician, is the principal investigator of the parent study, the LINCS UP project. The first author (UI) and several co-authors (NG, AS, HC) are LINCS UP co-investigators. While we bring extensive experience in substance use disorder (SUD) research, interventions, and service delivery, none of us have direct experience working as peer recovery coaches. As a result, our interpretation of peer recovery coach data may reflect a managerial or professional lens, potentially limiting insight into their day-to-day ED experiences. To address these biases, we actively involved the LINCS UP Community Advisory Board (CAB) and PRCs in reviewing and validating our findings. The LINCS UP CAB is comprised of service providers and program planners, most of whom have lived experience with SUD.

### Sample and recruitment

We interviewed the LINCS UP PRC team and program participants (ED patients with SUD served by LINCS UP), Grady ED physicians and staff, and representatives of local treatment and support services. LINCS UP program participants (patients) were referred to the study by PRCs serving these patients (PRCs had no role in the study enrollment or data collection). Other study participants (ED physicians, residents, nurses and social workers, and treatment and support service representatives) were invited to the study via email by the study PI (JC) and posters placed in the ED.

### Procedures

We conducted in-depth semi-structured interviews remotely via videoconference platform or telephone. Interview guides (see Supplemental File S1) were initially developed based on the literature on ED-based PRC program implementation and then reviewed and revised by LINCS UP CAB members: peer coaches, PRC managers and community partners (treatment and recovery service organization representatives, county health department representatives). Interview domains covered participants’ experience with the program; their opinions on program adoption, appropriateness and acceptability; key challenges and facilitators for successful implementation of the program; ensuring the quality of services; maintaining wellness of PRCs; and strategies and recommendations for improving PRC programs. We also asked participants to complete an anonymous online demographic survey. Participants provided verbal consent and received a $50 gift card as compensation for their time and effort. Emory University’s IRB approved the study protocol (STUDY00005344).

### Data analysis

We analyzed data using rapid qualitative analysis methods [19, 20]. Interviews were transcribed verbatim. We developed a templated summary table structured by key domains from the interview guide. Research assistants (RAs) (SA, TM, and TG) reviewed transcripts, summarized content, and populated tables with summary notes and illustrative quotes. The lead author (UI) reviewed the summary tables across the transcripts for consistency and accuracy of summaries. Under the lead author’s guidance, RA’s consolidated summaries into matrices by participant types and wrote memos identifying patterns, areas of convergence and divergence, and emergent themes related to implementation outcomes, strategies and recommendations. Data from the study participants was supplemented by LINCS UP leadership comments clarifying or providing additional information on changes in program implementation over time. We identified themes related to implementation outcomes based on Proctor et al.’s [17] framework. Specifically, we focused on themes related to appropriateness (e.g., perceived fit, relevance, and compatibility of the program to ED patient and staff needs) and acceptability (e.g., patients’ and providers’ perceptions of agreeableness, satisfaction and palatability of the program). We mapped specific examples of implementation strategies (see Table 2) to categories proposed by Leeman et al.’s [18] framework: (1) dissemination (informing and motivating stakeholders to adopt the intervention); (2) implementation process (planning and executing activities to adapt and implement the intervention); (3) capacity-building (strengthening implementers; general capacity to implement the intervention); (4) integration (addressing factors facilitating or hampering integration of the intervention into the practice) and (5) scale-up (strategies to implement an intervention across multiple settings). The study findings were reviewed by the LINCS UP research team, peer coaches and community advisory board members.

## Results

### Sample characteristics

We interviewed 27 program participants (patients) and 29 key contributors (9 ED physicians, 1 resident, 2 nurses, 4 clinicians, 4 social workers, 5 treatment facility representatives and 4 PRCs). In total, 52 of the 57 study participants completed a demographic survey. The program participant sample was predominantly male, Black and non-Hispanic. Among the 25 key contributors who completed the demographic survey, the majority were female, Black, and predominantly non-Hispanic. Both samples had an average age close to 43 years (Table 1).

**Table 1:** Sample characteristics (*N* = 52)

	LINCS UP participants (patients) (*n* = 27)	Key contributors (*n* = 25)
	n (%)	n (%)
Age, mean (SD)	43.4 (13.5)	43.1 (11.6)
Sex		
* Female*	7 (25.1)	15 (60.0)
* Male*	20 (74.1)	10 (40.0)
Race		
* Black/African American*	22 (81.5)	15 (60.0)
* White*	5 (18.5)	8 (32.0)
* Asian*	0	1 (4.0)
* No answer*	0	1 (4.0)
Ethnicity		
* Hispanic*	2 (7.4)	2 (8.0)
* Non-Hispanic*	23 (85.2)	23 (92.0)
* No answer*	2 (7.4)	0

### Implementation outcomes

We identified several themes related to the appropriateness of LINCS UP for ED patients and staff, such as successful linkages to community services, LINCS UP as an important resource for ED patients with SUD; added value of PRC team; and no or little negative impact on ED workflow by LINCS UP. We also mapped the theme of satisfaction with PRC interactions as an acceptability outcome. Description of these themes by implementation outcomes are provided below. We noted no differences or discrepancies between the in-person and telehealth arms of LINCS UP in terms of the implementation outcomes. Additional quotes are marked by a superscript letter and can be found in the Supplemental File S2.

### Appropriateness of the program

#### Successful linkages to community services

The LINCS UP program aims to engage patients with SUD and connect them to community-based services. Several patients shared their successes on their recovery journeys from SUD, including developing recovery plans^a^, joining in-patient treatment programs, and maintaining sobriety. Consistently, across program participants, patients reported feeling supported transitioning from the ED care team to local community-based resources. A LINCS UP participant recalled the process of linkage to a local SUD treatment program:

[PRC] reached out to a sober living house in the area, and she got me connected with them and actually got me a phone call interview with them. And I spoke with… I did the interview with [the program] and I was approved and accepted to the program, so [PRC] basically got me into the program, is what I feel like.

– A male program participant, age 27, interview 2

Another participant described how a PRC was constantly in contact with them during the process of recovery:

She was very kind to me, and she told me she was in recovery herself, and she told me I had what it takes to be living a sober life. She seen something. She was like, they will continuously help me throughout my whole process. And that’s what they did. That’s what they’ve been doing. I spoke with [PRC] yesterday. Like I said, I’ve been sober going on 60 days now. September 24th will be 60 days for me, and they call for call. Like, “Are you okay, […]? Anything you need to talk about? What you got going on?” [PRC team are] some great women.

– A male program participant, age 40, interview 1

A treatment facility representative also mentioned that LINCS UP participants have higher motivation and willingness to join the treatment compared to patients referred by other programs:

I will say this too, one of the key differences in referrals I get from y’all’s hospital system and from other hospital systems is that willingness. The individuals who I get as a referral or that we start the process with almost always end up coming. And that is something that not a lot of other places can say.

– A key contributor, interview 19

#### LINCS UP as an important resource for ED patients with SUD

LINCS UP participants shared that PRCs offered services that met their most urgent needs, including basic needs such as clothing, transportation, and referrals to treatment programs^b^. A LINCS UP participant described various services they received from a PRC:

*The peer coach*,* they actually made sure they provided me with a change of clothes and made sure I had adequate transportation once I leave. And they actually set me up to make sure I was*,* because I was transitioning into a program because I was homeless at the time and all. They set all that up for me. And they did the surveys for me and gave me the gift cards*,* which came in very*,* very helpful. So they was excellent. Yeah*,* it was right down my alley. It was what I needed at the time.*

– A male program participant, age 54, interview 31

Most ED providers and staff perceive LINCS UP and PRC team as an important part of holistic care for patients with SUD^c^. A mental health clinician emphasized the importance of the program:

*I think it’s a great program. It’s better than having those random papers that we pass out to them as an actual person*,* and they can do some peer counseling right there on the spot. So I think that’s really beneficial to the patients that we have here. And I do believe that it fits Grady’s roller skate environment because we are working in a roller skate environment here in the ER […]. It’s fast paced*,* and you have to be able to think your way through stuff.*

– A mental health clinician, interview 38

Representatives from local SUD treatment programs also expressed positive opinions about LINCS UP as a program serving people in need of SUD-related services in local communities, as shown in the quotation below:

So it’s servicing, probably, an underprivileged community, but also the servicing individuals in general that need and may want that additional help. They may not just want to stand still or stay still where they are. They want to move forward, but may not know how to. So you guys bridge that gap by connecting them with those resources, in order to further their treatment or stability.

– An SUD treatment program representative, interview 23

#### Added value of PRC team

Most interviewed ED staff and program participants agreed that due to lived experience, knowledge of SUD-related problems, and interpersonal skills, PRCs bring added value to patient care, eliminating stigma as barrier^d, e^. A nurse practitioner described how PRCs are effective in establishing rapport with the patients:

Yes, absolutely I think there’s added value. I think [PRCs] know how to talk to these patients in a way that they’re very invested and really do care. I just think they build really close rapport with these patients, the ones they can actually get through to, and I’ve seen it. […] I think there’s added value in maybe having that [lived SUD] experience, because they know how to create that safe place for the patient, communicate with them.

– A nurse practitioner, interview 42

#### No or little negative impact on ED workflow by LINCS UP

Most of interviewed ED staff did not report any negative impact from LINCS UP on ED workflow, such as delaying discharge or overcrowding the ED. A psychiatry intern also commented on the quick response time in the medical ED, but slower in the psychiatric emergency services area (PES).

What I do know is that in the PES, I frequently have to recall them just to make sure they’re coming. So it would have to be over 20 to 30 minutes versus in the ED. They were very quick. I never had to call and ask again for them to see the patient.

– A psychiatry unit intern, interview 22

However, one participant (an ED physician) mentioned that the LINCS UP process can impart a burden on the ED when patients with no acute health needs take up space in the ED that is already overburdened.

And these people who really don’t have any acute medical needs are generally sitting there for six, seven, eight hours while the peer recovery coaches figure something out, even if they see them relatively quickly. And then that bugs down the department, that’s a space that is not being used by a patient who has an acute medical need.

– An ED physician, interview 41

However, other ED physicians did not perceive shortage of space as a problem as they believe that there is enough space in the observation unit for patients desiring placement in a residential substance use treatment program:

From a space in the observation unit, we rarely have more than one or two people that are waiting on placement into a program. So, I feel like we generally have plenty of room there. So, I don’t feel like, honestly, it’s disruptive[…]. So, if anything, it helps flow by keeping patients out of the [emergency room] for problems that we are hopefully fixing or at least mitigating significantly.

– An ED physician, interview 43

### Acceptability of the program

#### Patients’ interactions with PRCs

Program participants are highly satisfied with their interactions with PRCs both in-person and remotely. Several participants mentioned feeling heard^f^ and comfortable interacting with PRCs. A LINCS UP participant described their positive experience communicating with a PRC in the telehealth component:

I realized [the peer coach] is somebody that I could reach out to when I can’t get to my therapist, my psychiatrist, my doctor, a friend, a neighbor, my mother and my father. I mean, I just know she’s a text away. She’s just a text away. It’s like, I don’t know how to explain it, that somebody else is taking on your worries and you don’t know if they have any worries. But she is great. I like her. We’ve never met, but just talking to her over the phone and seeing her face, I feel comfortable with talking to her about anything.

– A female program participant, age 54, interview 3

Patients’ positive experience with LINCS UP was in stark contrast with their previous experiences in other EDs where they were exposed to harsh and judgmental treatment^g^.

### Implementation strategies

We identified several themes related to implementation strategies summarized in Table 2 and described in detail further below.

**Table 2:** Overview of implementation strategies

Implementation strategy	Specific examples mapped to Leeman et al’s [18] classification (in parentheses)
*Streamlined communication between PRC and ED teams*	• Familiarizing ED providers and staff with the program and PRC team via presentations, flyers, ad-hoc meetings (Dissemination) • PRC and ED providers communicating via electronic medical records (EMR) system or a single phone number; updating ED providers and staff about clinical outcomes for individual patients via EMR (Integration) • Updating ED staff with program accomplishments (e.g., via newsletter) (Dissemination)
*Addressing barriers to community-based services*	• PRCs maintaining active communication with treatment facilities and other local service providers (Integration) • Timely provision of all medical and insurance documentation (Integration) • Finding services for uninsured or under-insured patients and assisting with transportation to treatment and other services (Implementation)
*Supportive supervision of PRCs*	• Daily team meetings to address daily operational issues combined with individual biweekly and annual meetings to discuss PRCs individual performance, challenges and opportunities for growth (Integration) • Comprehensive on-boarding orientation and on-job training; supporting PRCs mental health and addressing burnout by limiting caseload, restricting work outside of contract hours, providing wellness days-off (Capacity building)
*Addressing telehealth implementation challenges*	• Infrastructure for effective telehealth communication – iPads connected to stable Wi-Fi, HIPAA-compliant conferencing platform, headphones for privacy (Integration) • Ensuring access to EMR (directly or via staff stationed at a hospital) for telehealth PRCs who are not hospital employees (Integration)

#### Streamlined communication between PRC and ED teams

Streamlining the process of connecting ED providers with the PRC team to link eligible patients was an important strategy to ensure timely enrollment in the program. Most ED staff interviewed reported calling PRCs utilizing a designated phone number shown on contact cards placed around the emergency room^h^. In-person PRC team had access to EPIC, an electronic medical record system (EMR), where PRCs can access and input patient information.^1^ Some ED staff expressed their preference for using Epic to notify PRCs about a new patient (LINCS UP introduced this option subsequently). An ED physician found Epic chats more useful in reaching PRCs when calls weren’t being answered:

Typically, it’s been a very prompt reply, and capability to reach out to the patient, I usually contact [PRCs] through secure messaging through Epic…I think within the hospital setting, secure messaging through Epic or specific number to contact would be helpful. I think having a list of numbers is nice, but if it’s difficult to contact someone through those phones, people may be quick to give up and not try to contact them further.

– An ED resident physician, interview 39

Some participants also mentioned that employing a self-referral system or flagging patients with SUD earlier in the ED referral chain would be helpful to identify patients who would otherwise miss these services and link them to PRCs earlier in the referral chain^i^. An ED physician shared how self-referral or referral by non-physician staff may facilitate the timely linkage of patients:

I think if you rely on [ED physician] providers to make referrals, you would get a lot less uptake on that. I mean, people forget they would. So, I think allowing, having it be kind of a self-referral. Plus, or if the patient asks or if we are concerned or if the nurse is concerned or anyone’s on team’s concerned, you can call the number.

– An ED physician, interview 11

Familiarizing ED providers and staff with the program is the key to facilitating timely referral of patients. Many interviewed ED staff recalled learning about the program through flyers in the ED^j^ that have PRCs’ contact phone numbers^k^. PRCs also proactively introduce the program and themselves via planned presentations^I^ and ad-hoc meetings with the ED staff. An ED doctor remarked that PRCs are proactive in making their presence known in the ED:

The thing that’s probably the most awesome thing to me is that there are a lot of instances where the peer counselors are there before I even think to call them for a particular issue. So, they’ve been very proactive in just being in a department. They always come around in the morning and avail themselves to everybody, letting us know that they’re there, they talk to all of the providers.

– An ED physician, interview 17

Participants mentioned clinical communication between the PRCs and ED staff as an important strategy to facilitate program success. Timely communication between PRCs and ED staff is particularly important during shift changes for rotating staff, or when extending patients’ stay at ED before linking them to SUD treatment and other services. An ED physician explained that ED clinical staff should know if PRCs engaged the patients, provided any services or made any decisions:

I mean, for me, it’d be nice to know if it’s a patient I’m seeing for chest pain and the [PRC] talks to them, it would be nice to know that [a PRC] talked to them. So even if no services were provided or there’s nothing further that’s going to be required, almost like a quick note, “Hey, met with the patient. Offered these services. Here’s the plan from here.” They wouldn’t need to necessarily say anything verbally to the care team, but it’s just nice to know when you’ve got consultants essentially working with your patient.

– An ED physician, interview 11

Many participants expressed their desire to learn about LINCS UP’s successes and outcomes^j^. An ED physician suggested receiving regular updates via a newsletter to inform ED staff of the program accomplishments (this recommendation was eventually implemented by the program):

I think probably a regular newsletter communication. Maybe that showcases the number of patients seen and kind of the breadth of services that you guys offer. I mean, we talked about it, Joe and I talked about it a little the other day actually. I didn’t realize that there’s been what? 700 plus patient contacts. And I think some people would like a vignette, a story of if the patients are okay with that. Share a story of, “Hey, this person went to these services” or “This person got placed in a rehab facility and is doing well” or something like that.

– An ED physician, interview 11

An ED physician mentioned wanting to be informed on how to safely engage patients in a conversation about substance use in a productive and respectful manner:

I think just more information on how to safely engage patients in these conversations about substance use, where we’re not inadvertently committing errors that can harm the relationship or that could impact whether or not they’re willing to engage us. That’s what I would really like to learn the most.

– An ED physician, interview 17

#### Addressing barriers to community-based services

LINCS UP participants appreciated the referral process, which addressed barriers to linkage and enrollment in treatment programs, such as PRCs calling local service providers to find vacant spots, assessing patients’ eligibility for services, preparing the package of documents and arranging transportation. One program participant mentioned that LINCS UP referral process, unlike their previous experience with ED, ensured successful linkage to services.

I’ve been in this situation before. So, there was a time that I had a case worker come see me. And I think that was the only time I saw her, and that was the last time I saw her. I gave out information concerning the issues that I had, but I never received any type of hands-on care. I wasn’t connected to anyone, other than being told that I need to meet my appointments with Grady. So, this time I was actually put in contact with the director at the recovery center. And I was accepted into the program, as well as being able to have transportation to get to the program. So, that was really hands-on.

– A male program participant, age 56, interview 6

Communication and timely provision of patient documentation are key for successful referrals. A treatment facility representative explained that the process of requesting additional medical documentation, including lab tests, may delay the process of linkage and recommended sending all relevant medical information with the LINCS UP participant (this problem was subsequently addressed by LINCS UP):

One thing I did notice is that we have a lot of people come, and we have to try to dig for extra information we don’t want to. We kind of have to get it from the [LINCS UP participant] because of client confidentiality. So I don’t want to go and say, “Hey, can you send this over? Do you have their medical documents or any of their medical records?” If there’s anything extra that the client can be left with or in an envelope or what have you that lets us know. Have they had TB screen, [syphilis screening]? That’s excellent to know because that’s two steps faster closer to getting them into treatment.

– An SUD treatment program representative, interview 18

LINCS UP effectively addressed transportation as a barrier to services. Most treatment programs offer transportation for the patients from the hospital to the program, in other cases LINCS UP uses car sharing services funded by the hospital^m^. A local SUD treatment program representative commended LINCS UP team for ensuring patients are seamlessly transported to the program:

*I love how they sit with them until that ride shows up for them*,* whether it’s an Uber or a transporter*,* that they’re actually waiting for the ride to show up with the individual*,* because sometimes someone gets discharged from a hospital and they kind of vanish. […] A lot of individuals don’t have phones. And I run into problems [reaching the patients] sometimes*,* so I love that they do that.*

– An SUD treatment program representative, interview 25

Most SUD treatment programs require health insurance which can be a barrier for some patients. While these programs cannot accept participants without health insurance, they do often help with the referral to other facilities^n^. A treatment facility representative described how LINCS UP staff ensure that patients insurance information is up-to-date and provided to the program:

I would say, this is one that I mentioned before, which is the access to documentation is a barrier. Not so much for me, because they’re always able to get it to me, the team there at LINCS UP. But I know sometimes that they have to run across and get a member ID from an insurance person, or someone’s insurance if it’s not in the system.

– An SUD treatment program representative, interview, 19

#### Supportive supervision of PRCs

PRC team leaders oversee day-to-day operations and provide long-term support for PRCs well-being and performance. Team leaders mostly interact with PRCs via several types of meetings with various scope and frequency, such as morning huddles, bi-weekly supervisory meetings and annual performance reviews. Morning huddles are daily team meetings held to distribute assignments, track the workload and address daily operational issues. During bi-weekly meetings, supervisors meet individually with each PRC to discuss ongoing challenges, provide performance feedback, and support their well-being. Annual evaluations offer an opportunity to review PRCs performance over the longer term, discuss accomplishments and opportunities for professional growth and development. In addition to scheduled meetings, the PRC team leader maintains communication with PRCs via phone and e-mail to help resolve conflicts or provide recommendations on engaging patients.

PRC participants emphasized the importance of on-job training, including orientation for newly hired PRCs to familiarize them with the ED and hospital environment, structure, and procedures. Lack of structured orientation for PRCs with no healthcare background may impede effective onboarding, as one PRC shared:

[…] the certification to become a peer specialist is [just] a 40-hour class. And so if you have peers come into the role, they may or may not have experience in healthcare, they may or may not have experience working with peers, but I had to learn all of this on my own. I didn’t really have a whole lot of structural support for, here are the parameters of the program, here’s how we write notes, here are the inner workings of a hospital. All those things I had to just learn on the day to day, if that makes sense?

– A peer recovery coach, interview 52

LINCS UP employs various strategies to support PRCs’ mental health. PRCs have access to counselling and can take one wellness day off every quarter in addition to paid leave. The program also addresses burnout by limiting PRCs’ caseload and restricting work outside of the official working hours. However, high demand for PRC services at the ED coupled with the lack of resources to hire additional PRCs adds workload for who may be reluctant to deny services to patients in need even when PRCs capacity is limited. High workload may also prevent PRCs from taking wellness days off since other PRCs will have to uptake additional patients or deny services to them, which may further exacerbate PRCs mental health. A PRC explained the reasons for not taking wellness days off:

Yes. I know that we have allotted wellness days. That said, have I ever taken one? No. Well, I’ve taken half of one. […] Because you feel that if you take them, then you’re leaving all of your peers and your work for someone else to do, and so then you feel guilty and you don’t… I’ve avoided it because I know if I leave that [another PRC] is going to have to take not only her peers, but all of mine.

– A peer recovery coach, interview 52

PRCs may also take a short break after a challenging encounter with a patient, as a PRC mentioned:

…if we’ve had an encounter and we feel like we need a minute, we’re able to walk away and just let the other team members know like, ‘Hey, I need a break for a second.” And then we also get two 15-minute breaks.

– A peer recovery coach, interview 53

#### Addressing telehealth implementation challenges

PRC team members discussed challenges related to implementing telehealth PRC program and strategies to address them. A key challenge was that PRCs in telehealth arm were not hospital employees and therefore lacked access to the hospital’s EMR (in contrast, in-person PRC team had access to EMR). This problem was solved by having an in-person PRC team member assist the telehealth team with documenting their recommendations in the EMR and relay EMR messages back to them. PRCs used a secure conferencing application (RingCentral) to communicate notes about the patients between the in-person and telehealth teams. However, this workaround may lead to confusion and delays in communication, as explained by a PRC team member:

For example, [telehealth PRCs] don’t have [EMR] access so in addition to my workload, I also have to assist with getting [telehealth PRCs] on the phone with peers, input their notes, which has particularly been a challenge because they’re not inputted, they’re not sent over in an adequate amount of time and so then notes go in late. But because I sign off of them, it reflects poorly on me, even though they’re not my peer.

– A peer recovery coach, interview 53

PRC team lead mentioned that obtaining full access to EMR for the telehealth team would be the optimal solution:

I lie to you not, it would be great if we had Epic access. So that would be a direct conversation with doctors and the actual peer coach, because it wouldn’t be a third party in there.

– A PRC team lead, interview 51

As noted by LINCS UP leadership team, telehealth PRC team lacked opportunity to interact with the hospital staff face-to-face, limiting the former’s ability to establish informal professional communication with the hospital staff. To address this challenge, the program distributed flyers to the hospital staff introducing both in-person and telehealth PRC team. In-person PRCs also assisted in introducing and connecting telehealth PRCs with the hospital staff. Further, telehealth PRCs experienced difficulties connecting with the ED doctors and staff via phone. This problem was solved by in-person PRC team members helping the telehealth team to contact ED staff.

Infrastructure-related telehealth implementation strategies included providing the program participants (patients) iPad tablets connected to the hospital’s stable, secure Wi-Fi network and conferencing platform for telehealth sessions. By employing standardized, commercially available hardware and software familiar to the team, potential technological barriers were effectively mitigated, enabling efficient and streamlined implementation. The program has also provided headphones to patients to ensure their privacy since securing a private area for telehealth sessions was not always possible.

## Discussion

ED-based PRC programs are increasingly being implemented by health system leaders as effective interventions linking patients with SUD to hospital and community-based services. This program evaluation study has identified themes related to implementation outcomes and strategies of an ED-based PRC program serving patients with SUD in a major hospital in the US South. These themes illustrate various aspects of acceptability and appropriateness of LINCS UP as program implementation outcomes. Themes related to implementation strategies, including timely linkage of patients to PRCs, effective communication between PRC and ED teams, addressing barriers to community-based services, supportive supervision of PRCs and addressing telehealth implementation challenges provide specific recommendations for ED-based PRC program implementers. Collectively, these findings can guide other health systems in implementing PRC programs to improve linkages to care for individuals living with SUDs.

Our data show that PRC programs appropriateness as an implementation outcome may have several facets. Specifically, the participants agreed that the program has been successful in both addressing patients’ immediate needs and then linking them to community-based services. These two components represent a core function of ED-based PRC programs [21]. Most participants, both ED staff and patients, agreed that PRCs – individuals with lived experience – bring added value to services for patients with SUD. Literature emphasizes that the substance use and recovery experience which is shared by PRCs and patients is a unique characteristic contributing to PRC program success [8]. Finally, participants reported that LINCS UP had little or no negative impact on ED operations and patient workflow. The majority of staff, including physicians, nurses, and other clinical team members, found the PRC program to be a benefit to improving care services for individuals presenting in the ED with a SUD without disrupting clinical workflow. This finding may help alleviate concerns that PRC programs may disrupt ED services or extend patient stay thereby incurring additional costs.

Implementation strategies identified in our study might be helpful for other ED-based PRC programs. Effective communication between PRC team and ED providers is key for program success, particularly regarding timely linkage of patients to the PRC team. We identified several approaches to effective clinical communication, such as streamlining the process of ED staff informing the PRC team about new patients via designated phone number or the EMR system; receiving timely feedback from PRCs to ED providers on patient’s engagement and linkage to services. ED staff should be familiar with the PRC team’s role, their functions and program objectives; and aware of programmatic aspects of the program, including information on the program accomplishments and success stories. Miscommunication or lack of communication between PRCs and ED clinicians can lead to confusion about the scope of PRC roles and impede patient service [22].

Streamlined clinical communication between PRCs and clinical providers is essential for telehealth PRC programs. Ideally, telehealth PRCs should be able to communicate with ED providers directly via EMR or other secure messaging systems. If direct clinical communication is not possible, a specific hospital contact should facilitate clinical data entry and retrieval from EMR (the approach eventually undertaken by LINCS UP for telehealth component). This indirect access to EMR might be the most feasible solution for PRC telehealth teams that might cover multiple hospitals and/or hospital systems, since these PRCs likely would not have access to each system’s EMR. Programs should introduce telehealth PRC teams to hospital clinical providers and staff, including, if possible, regular in-person meetings or presentations by telehealth PRCs. Ensuring proper infrastructure for telehealth sessions, including provision of handheld devices with stable Wi-Fi connection and headphones for patients’ privacy, and HIPAA-compliant conferencing platform is also an important implementation strategy for telehealth PRC programs.

Effective linkage of patients to community-based programs may face numerous barriers. Our study identified several approaches to facilitate linkage of patients to services. These approaches include establishing effective communication between PRCs and staff of SUD treatment facilities and other service providers, ensuring patients’ eligibility for community-based services, assisting patients throughout the linkage process, preparing all documentation and information necessary for admission to services, and finding services accepting uninsured or underinsured patients and providing transportation. Linkage to community-based services, including behavioral therapy, is especially important for patients with stimulant use disorder in the absence of effective medication-based treatments for this condition [23, 24]. A proactive approach is a key to successful linkage of patients with SUD to community-based services [25, 26].

We identified an important set of recommendations related to organizational and structural support for PRCs. Supportive supervision may take various forms, including meetings of various frequency and scope (e.g., daily, bi-weekly, ad-hoc and annual) to maintain effective communication between PRCs and program leadership, identify and address problems and provide guidance and support to PRCs. Supportive and recovery-oriented supervision is essential for maintaining quality of services, protecting PRC mental health and preventing burnout [7, 9]. Other approaches, such as wellness days off, on-job counselling and limiting PRC workload also play a critical role in ensuring PRCs mental health [15].

Finally, in addition to eliciting specific implementation strategies, this study shows the program adapted its implementation and services in response to program partners’ feedback. We documented several occasions of LINCS UP program adaptations, including clinical communication via EMR, circulating the program newsletter among ED staff, and timely preparation of documentation for referrals. These findings show how on-going process evaluation and program adaptations may facilitate successful implementation of a PRC program.

Our study has several limitations. We present the results of evaluation of one program implemented in a major city hospital ED, so our findings may have limited generalizability to EDs in smaller or rural areas. None of the study team members involved in data collection have worked as PRCs or have SUD recovery experience, which may bias our understanding and perception of data. Nevertheless, given that we interviewed a wide range of participants, including individuals with SUD who received program services and various service providers from ED setting and form the community, our findings might be valuable for other ED-based PRC programs. This study’s large, diverse sample size, relative to other qualitative investigations [27], improves upon previous implementation evaluations of PRC programs and provides recommendations from the perspective of patients, PRCs, and clinical staff to guide successful implementation.

## Conclusions

The use of PRC programs in ED settings to address the needs of patients presenting with SUD are demonstrating promise. Successful adoption and implementation of a PRC program in such a setting is dependent on addressing a wide range of barriers. Our study describes the program implementation outcomes and presents a set of strategies to ensure program success.

## Supplementary Information

Below is the link to the electronic supplementary material.

Supplementary Material 1

Supplementary Material 2

## Data Availability

The qualitative dataset generated and analyzed during the current study is not publicly available to protect participants’ confidentiality. Anonymized summaries of transcripts can be made available upon reasonable request and signing of data sharing agreement.

## Footnotes

1 Telehealth PRCs did not have direct access to EMR - see sub-Sect. 3.5.4*Addressing telehealth implementation challenges* for more details.
