# Correction: Implementation outcomes and strategies of a peer recovery coach program: findings from a qualitative assessment in the U.S. South, 2024–2025

**DOI:** 10.1186/s13722-026-00648-4

**Published:** 2026-01-21

**Authors:** Umedjon Ibragimov, Nicholas A. Giordano, Sneha Amaresh, Tatiana Getz, Tatiana Matuszewski, Alaina R. Steck, Yan Li, Eliot H. Blum, Jessica Tuttle, Hardik Pipalia, Hannah L. F. Cooper, Joseph E. Carpenter

**Affiliations:** 1 Center for Population Sciences and Health Equity, College of Nursing, Florida State University, 2010 Levy Avenue, Innovation Park, Building B, Suite 3600, Tallahassee, FL 32310 USA; 2 Nell Hodgson Woodruff School of Nursing, Emory University, Atlanta, Georgia; 3 Department of Behavioral, Social and Health Education Sciences, Rollins School of Public Health, Emory University, Atlanta, Georgia; 4 Department of Emergency Medicine, School of Medicine, Emory University, Atlanta, Georgia; 5 Georgia Department of Public Health, Atlanta, Georgia; 6 Aniz, Inc. Holistic Harm Reduction Integrated Care Clinic, Atlanta, Georgia; 7 Georgia Poison Center, Atlanta, Georgia

**Correction: Addict Sci Clin Pract 20, 95 (2025).**

10.1186/s13722-025-00624-4.

Following the publication of original article [1], it is reported that there was a mistake in a co-author’s name: Hardick Pipalia should be corrected to Hardik Pipalia.

The original article [1] has been corrected.
