# Supplementary material for: Implementation outcomes and strategies of a peer recovery coach program: findings from a qualitative assessment in the U.S. South, 2024–2025
Source: Addict Sci Clin Pract. 2025 Dec 18;20:95. doi: 10.1186/s13722-025-00624-4 (PMC12713283; doi:10.1186/s13722-025-00624-4)
Supplement: Supplementary file 1 — Supplementary Material 1 [file 13722_2025_624_MOESM1_ESM.docx]

**Supplemental file S1.**

**SEMI-STRUCTURED INTERVIEW GUIDES**

*Wording, probing questions and the order of questions may change depending on the interview or FGD flow*

**Individual interview guide for PRC program participants**

1. Please tell me how did you end up at Grady emergency department.
   1. What brought you to ED?
   2. Who accompanied you to Grady?
   3. Who came to visit you at Grady?
   4. How long did you stay at Grady?
   5. How was your experience working with the ED staff?
2. What were your thoughts when you were invited to join PRC program at Grady?
3. What was the process of joining PRC for you?
   1. Was this process easy, or is there something that can be changed to make it easier or more convenient (i.e. challenge with follow-up phone contact, etc.)
   2. What did you like or didn’t like about it? Why?
4. Tell us about your experience with PRC.
   1. What were your major needs, problems, and concerns at that time?
      1. Did you tell peer coaches about all your problems or needs?
   2. What services or supports did peer recovery coaches connect you to at Grady?
      1. What did you like or dislike about these services? Why?
   3. What needs or problems you couldn’t get support with? Why?
      1. If you still have these problems, how are you addressing them?
5. What were your impressions of the peer recovery coach (PRC)?
   1. What did you like about them? What didn’t you like? Why?
   2. What was the most memorable moment of working with the peer coach for you? Why?
   3. How did your relationship with the peer recovery coach change over time?
6. Tell us about other PRC staff (not peer coaches) that worked with you.
   1. How helpful were they?
   2. What did you like or didn’t like about them? Why?
7. If you were connected to other organizations outside of Grady, tell us about it.
   1. Which organization(s) were you linked to?
   2. What kind of support did you need at that point?
   3. What did you like or didn’t like about this process of being connected to other organizations?
   4. What made it easy or hard to get support from those organizations?
      1. Time, transportation, staff attitudes, service hours, location, costs, insurance.
8. If PRC helped you with getting to a treatment program, tell us about it.
   1. How long have you stayed in treatment?
   2. What made it easy or hard to get into treatment?
9. How was PRC helpful in connecting you to overdose prevention?
   1. Naloxone
   2. Education
   3. Fentanyl test strips
10. What other SUD or harm reduction services did you get?
    1. Access to clean syringes and other injecting equipment.
    2. HIV and Hep C testing
    3. HIV and Hep C treatment
    4. PrEP
11. What about services like housing or employment?
12. How else did PRC help improve your life?
13. What PRC couldn’t help with? Why?
14. How PRC can be improved?
    1. What do you think we should change about the way peer coaches work with you?
    2. What should be changed about services or type of support you can get at Grady?
    3. What should be changed about the way you are connected to organizations outside of Grady?
       1. What about connecting to SUD treatment – how can it be improved?
       2. What can we improve about overdose prevention?

**An interview guide for peer recovery coaches**

1. What is your role as a peer coach in supporting patients with SUD?
   1. What kind of patient assessments do you conduct?
   2. What specific services do you provide for individuals with substance use disorder?
   3. How often and how long do you interact with the patients?
      1. While patients are in the ED
      2. After they are discharged?
2. What kind of support do you receive to make sure you are providing quality services to patients?
3. On-job training
   - 1. By whom, contents, modules?
4. Mentorship
   - 1. Who is the mentor?
5. Working with SUD-related problems may be stressful. What kind of support is important to prevent chronic stress and burnout of peer coaches?
   1. How does the workload affect the quality of services?
6. What is the role of the PRC supervisor in ensuring PRC’s wellness and quality of services?
7. What other types of support or resources are needed for effective work of peer coaches?
8. How do you interact with hospital staff while serving the patients?
   1. Who, what kind of interaction, data sharing
   2. What kind of support or assistance is provided by the hospital staff
   3. How can this interaction be improved?
9. What services inside and outside the hospital are the patients linked to?
   1. Which ones do you hear back from/patients find most useful?
   2. Why do you think that is?
10. How do you interact with community-based organizations while serving the patients?
    1. Who, what kind of interaction, data sharing
    2. What kind of support or assistance is provided by the community-based organizations staff?
    3. How can this interaction be improved?
11. What problems have you had with linking patients to community services in the past or currently?
    1. How were these problems solved?
       1. Alternatively, what suggestions do you have or solutions that may overcome these problems?
12. What other lessons learned or recommendations would you like to share with us?

**Interview guide with Grady staff (doctors/nurses/social workers)**

1. What is your role in serving patient with SUD?
2. Tell us about your experience with PRC.
   1. How did PRC change the way you work with the SUD patients?
3. What is your opinion of PRC?
   1. How does PRC fit Grady priorities?
   2. How does PRC meet patients’ needs?
4. What problems did you have or anticipate with PRC?
   1. Workload, clarity about rules and procedures, staff or patients’ safety.
   2. Who did you communicate these problems to? If not, why? If yes, what happened?
5. What should be done to solve these problems?
   1. What can you do to help solve these problems?
6. How LINCS UP can be more effective in preventing violent injury?
   1. What can you do to make it more effective?

**Interview guide for community partners**

1. What is the role of your organization in serving people with SUD?
2. Tell us about your experience with PRC.
   1. How did you decide to work with PRC?
   2. What do you like about PRC? Why?
   3. What don’t you like about PRC? Why?
   4. How does PRC fit your organizations’ mission and priorities?
   5. How did PRC change the way you work with people with SUD?
3. What do you think about PRC effectiveness?
   1. How does PRC meet clients’ needs?
      1. How does PRC help to link patients to drug treatment? Overdose prevention? HIV and Hep C services?
      2. How does PRC help to link patients with SUD to social services (housing, employment)?
4. What can be done to improve PRC effectiveness in preventing overdoses? Reducing other drug-related harms such as HIV, Hep C?
5. How did PRC change the way you work with Grady? With other organizations or agencies?
   1. What do you think of the process of Grady patients’ referral to your organization?
6. What is the process of communication and data exchange or sharing within PRC?
   1. How it is different from what was before? What challenges remain?
7. What challenges or problems did your organization have with PRC?
   1. Staff workload, communication, resources, funding, staff or clients’ safety.
   2. What was done about these problems? If nothing, why?
8. Can you continue these services without funding from Grady or CDC?
9. What needs to be done to ensure these services are sustained after PRC funding ends?
