# Supplementary material for: Implementation outcomes and strategies of a peer recovery coach program: findings from a qualitative assessment in the U.S. South, 2024–2025
Source: Addict Sci Clin Pract. 2025 Dec 18;20:95. doi: 10.1186/s13722-025-00624-4 (PMC12713283; doi:10.1186/s13722-025-00624-4)
Supplement: Supplementary file 2 — Supplementary Material 2 [file 13722_2025_624_MOESM2_ESM.docx]

**Supplemental file S2. Additional quotes**

*letters in Column 1 indicate superscript symbols referencing additional quotes in the article

| ***Successful linkages to community services*** | |
| --- | --- |
| a* | "Participant: I did a relapse prevention plan, or I think that's what it's called, I did that with her over the phone. It took an hour, hour and a half, and she asked me a whole bunch of questions and wrote it down, and I have a copy of it emailed to me.  Interviewer: And how was that? What were your thoughts on that?  Participant: Some of them was kind of touchy and sensitive for me, but then it really made me realize if a situation occurs, you really do need to plan. Because I was thinking in my head when we were doing this, I was like, "Oh my God, if this happens, I'm not going to think to do this and that." But if you really think about it and talk about it out loud, it is good to have a plan instead of just winging it, because when feelings are involved, then it's harder. So it was really nice to have that and think about it*.*  -LINCS UP program participant, female, age 39, interview 27 |
| ***LINCS UP as an important resource for ED patients with SUD*** | |
| b | [The peer recovery coach] basically just explained that she was there for support in my recovery, and she had a lot of resources and stuff that she could help me with.”  A male program participant, age 27, interview 2 |
| c | I think it's a great match and very much needed program. Yeah, absolutely. So many of the patients we see in PES, there's substance abuses, comorbid, or primary, so it's indispensable.   - A licensed clinical social worker, Psychiatry Emergency Services, interview 20 |
| ***Added value of PRC team*** | |
| d | I think that connection [between PRCs and patients] makes a tremendous difference. When you can connect someone on a very personal level, I feel like it makes them feel not so alone because a lot of social stigma that can go with a lot of substance abuse disorders and saying like, "Hey, I get where you're coming from. I've been in similar shoes," and I feel like that speaks volumes to the person like, "Yeah, you truly do," because me as a physician I can say, I understand, but I don't right? I haven't been in your shoes before. So I think that's of tremendous value. That said, there may be additional set of expertise when it comes to social workers, for example, in terms of understanding the system. So I think it's probably, and as it is a at least partially multidisciplinary approach. The peer recovery coaches can speak on a very personal level to the patient, but then filtering in people who potentially can understand the resources and the opportunities that are out there for patients is also a pretty good value.   - An ED physician, interview 43 |
| e | It was very nice. They asked me first. Would I... Asked my permission to speak to me, and I said, yes. They gave me their business card. I was in a messed up situation, because I didn't have no shoes at the time on my feet. The lady got up and went and found me a pair of shoes out of the stock, what they had at Grady. She was very kind to me, and she told me she was in recovery herself, and she told me I had what it takes to be living a sober life. She seen something. She was like, they will continuously help me throughout my whole process. And that's what they did.  -LINCS UP program participant, male, age 40, interview 1 |
| ***Patients’ interactions with PRCs*** | |
| f | Someone actually came and talked to me and actually listened to what I had to say and actually helped me. Because usually I went to the hospital before with suicidal ideations and they just want to send me to a behavioral center type thing. And then when I get released from there, it's the same thing, I'm back on the streets.  – LINCS UP program participant, male, age 27, interview 2 |
| g | I can't think of anything. I do want to say that hands down, Grady has been the best because this is not my first overdose and I've always went to Cobb or Douglas, because that's the area that I live in. But at the time I was in the area of Grady when I overdosed and the way I was treated, the stay, the seriousness of it, the non- judgmental, the doctors, the aftercare, the peer specialists, none of that's offered anywhere else. You ask for help somewhere else, none of that is available. I feel like it's just you're looked down upon for having an addiction and it was not like that at Grady at all. And it was really great to experience a different experience. And when people don't treat you like that, it makes you want to do better instead of just like, "Well, fuck it." So…  -LINCS UP program participant, female, age 39, interview 27 |
| ***Streamlined communication between PRC and ED teams*** | |
| h | In the instances where I do need to reach out to them, we have little place cards distributed throughout the ER that I'm able to contact them as needed. So, it's been I think pretty easy in terms of reaching them when I do need them or that I think they could be helpful in a case.  – An ED physician, interview 17 |
| i | I mean I feel like, I know I miss patients that might benefit from talking to them, but it's something that in light of, like I said, all of the other things that we're dealing with from the medical standpoint or trauma is something that can get glossed over and we might miss an opportunity with folks. And maybe it doesn't have to be in triage, but somewhere along the nursing, even ancillary staff might be able to help us highlight who might be the folks to engage and that would optimize their outcomes, I think that would be useful.  – An ED physician, interview 17 |
| j | Maybe the team members came by the PES and we have the flyers around the unit now, so I don't exactly remember how I was introduced to them though.   - A licensed clinical social worker, Psychiatry Emergency Services, interview 20 |
| k | …there's so very many [informational] flyers on our [ED] walls, which speaks to like you have to know the program already exists and then be like, "Oh yeah, that's where the flyer is." And I think that surprisingly works for most people. But again, if you didn't know about the program, if you were just like, go look at this wall, you'd be like, I don't know, this is a bunch of stuff. So I feel like that isn't a very effective means of communication, but it's a good means of figuring out how to get in touch with them. It's very easy. It's posted in, at least I looked at it in trauma yesterday and I'm pretty sure it's in zone two and three as well.  *-* An ED physician, interview 43 |
| l | I believe that some of the key representatives of LINCS UP gave us a presentation on what LINCS UP is and a little bit about how it works and who they are. So that is how I learned about what the organization is and what they wish to accomplish.”   - An ED resident physician, interview 39 |
| j | I think in terms of larger communication, I think it'd be really good [for LINCS UP] to have a follow-up with the Grady faculty. I don't know if this exists at Emory or Midtown [hospitals], so I'm only speaking to Grady, but having a follow-up with the Grady faculty saying, "Hey, this is an overview. In 15 minutes, this is where we started. This is where we are. This is the impact we've made. These are our future directions." I think it's really cool for people to see wins like that. So I think that would be really cool to know. And I think it'd be interesting also, and this may not be in the same format, but to learn, maybe have a spotlight if the peer recovery coaches are willing to share their story to us, I think that's a really meaningful experience too. But I understand that's their personal business, so only if they would want to, but I think that'd be a really interesting thing and really puts a face behind a program.  -An ED resident physician, interview 39 |
| ***Addressing barriers to community-based services*** | |
| m | Once again, usually if there was an issue or anything, it was usually addressed right then. Like I said, with one client, we had an issue with trying to get them here properly. And that's when they started coming in Lyfts and Ubers, and by train. They were able to start getting here.   - A local mental health emergency service representative, interview 18 |
| n | I feel insurance is probably just the biggest barrier for our program, but again, we have some solutions and some steps that individuals can take in order to either come to our program or if maybe another program is a better fit.   - An SUD treatment program representative, interview, 19 |
